# Predictors and Prognostic Impact of In-hospital Bleeding after Transcatheter Aortic Valve Replacement According to BARC and VARC-2 Definitions

**DOI:** 10.21470/1678-9741-2019-0275

**Published:** 2019

**Authors:** Adrian daSilva-deAbreu, Yelin Zhao, Astrid Serauto-Canache, Bader Alhafez, Katyayini Aribindi, Prakash Balan, Pranav Loyalka, Biswajit Kaaaar, Richard Smalling, H. Vernon Anderson, Abhijeet Dhoble, Timo Siepmann, Salman A. Arain

**Affiliations:** 1 Department of Internal Medicine, McGovern Medical School, The University of Texas Health Science Center at Houston, Texas, United States of America.; 2 Division of Health Care Sciences, Center for Clinical Research and Management Education, Dresden International University, Dresden, Germany.; 3 Ochsner Clinical School, Faculty of Medicine, The University of Queensland, New Orleans, Louisiana, United States of America.; 4 Division of Cardiovascular Disease, Department of Internal Medicine, McGovern Medical School, The University of Texas Health Science Center at Houston, Texas, United States of America.; 5 Department of Neurology, University Hospital Carl Gustav Carus, Technische Universität Dresden, Dresden, Germany.

Dear Editor,

Bleeding after transcatheter aortic valve replacement (TAVR) is associated with prolonged hospitalization and mortality^[1,2]^. Most TAVR studies in the United States of America (USA) only report 30-day and one-year outcomes using the definition of The Society of Thoracic Surgeons/American College of Cardiology Transcatheter Valve Therapy (STS/ACC TVT) Registry, which is a simplified version of the Valve Academic Research Consortium 2 (VARC-2) scheme that only dichotomizes the presence or absence of bleeding without stratifying its severity^[3,4]^.

Although few studies have evaluated the relationship between severity of bleeding and postprocedural outcomes according to both VARC-2 and Bleeding Academic Research Consortium (BARC) bleeding definitions^[5]^ at 30 days and one year after TAVR, they did not investigate in-hospital bleeding (IHB) events^[1,2]^. Hence, there is paucity of information about post-TAVR bleeding events during index hospitalization, as well as their impact on outcomes, especially in widespread clinical practice and not just in clinical trials.

We conducted a retrospective cohort study using the TAVR database of the Memorial Hermann-Texas Medical Center, one of the largest TAVR centers in the USA. We included all patients who underwent TAVR between November 2011 and December 2016, using the STS/ACC TVT criteria to identify postprocedural IHB events. A multivariate logistic regression was performed to identify clinical characteristics predictive of IHB. Pearson’s chi-square test, logistic regressions, and Wilcoxon rank test were used to analyze the association between the presence and severity of IHB, according to BARC, STS/ACC TVT, and VARC-2 classifications and the following outcomes: in-hospital mortality, 30-day mortality, length of stay (LOS) (in days), intensive care unit (ICU) LOS (in hours), and discharge location (home *vs*. intermediate facility [nursing/rehabilitation facilities, funeral home, etc.]). All statistical tests were performed with Stata SE 14 software (StataCorp, College Station, TX). A *P*-value < 0.05 was considered statistically significant.

Among the 1,036 patients that underwent TAVR, 115 (11.1%) developed postprocedural IHB. Sources of bleeding were the access site (28.5%) and retroperitoneal (16.9%) and pericardial (12.3%) tamponade. Serum albumin level (odds ratio [OR] 0.61 per gram of albumin), hemoglobin level (OR 0.87 per gram of hemoglobin), transaortic access (OR 4.1), and conduit access (OR 4.8) were independent predictors of IHB risk. Patients with IHB experienced higher risk of in-hospital mortality (15.7% *vs*. 1.2%), 30-day mortality (14.8% *vs*. 2.1%), vascular complications (20.9% *vs*. 3.7%), longer LOS (10 *vs*. 5 days), longer ICU LOS (124 *vs*. 46 hours), and higher risk of being discharged to an intermediate facility (47.8% *vs*. 14.7%) than patients without IHB. The incidence of these complications increased with the severity of bleeding by both BARC and VARC-2 criteria (Table 1). All *P*-values were < 0.05.

**Table 1:** Postprocedural outcomes of patients undergoing TAVR according to their IHB complications per BARC, STS/ACC TVT, and VARC-2 classifications.

Outcomes	TVT		*P*-value	BARC*				*P*-value	VARC-2			*P*-value
	No bleeding	Bleeding		Type 3A	Type 3B	Type 3C	Type 5		Minor	Major	Life-threatening/Disabling	
	n=921	n=115		n=38	n=62	n=3	n=12		n=17	n=27	n=71	
In-hospital mortality	11 (1.2)	18 (15.6)	<.001	2 (5.3)	3 (4.8)	1 (33.3)	12 (100)	<.001	2 (11.8)	0 (0)	16 (22.5)	<.001
30-day mortality	19 (2.1)	17 (14.7)	<.001	2 (5.3)	3 (4.8)	1 (33.3)	11 (91.6)	<.001	2 (11.8)	0 (0)	15 (21.1)	<.001
Length of stay (days)	5±6	10±9	<.001	8±8	11±8	21±2	10±11	<.001	8±8	8±8	12±9	<.001
ICU length of stay (hours)	46±73	124±128	<.001	86±100	128±115	373±265	162±160	<.001	93±130	76±72	150±138	<.001
Discharge to intermediate facility†	136 (14.7)	55 (47.8)	<.001	11 (28.9)	29 (46.8)	3 (100)	12 (100)	<.001	3 (17.6)	10 (37)	42 (59.2)	<.001
Vascular complications	34 (3.7)	24 (20.9)	<.001	7 (18.4)	14 (22.6)	0	3 (25)	<.001	2 (11.8)	7 (25.9)	15 (21.1)	<.001

* Discharge to intermediate facility included nursing or rehabilitation facilities and funeral home. Values in n (%) or mean ± standard deviation.

† Discharge to intermediate facility included nursing or rehabilitation facilities and funeral home. Values in n (%) or mean ± standard deviation.

BARC=Bleeding Academic Research Consortium; IHB=in-hospital bleeding; STS/ACC TVT=The Society of Thoracic Surgeons/American College of Cardiology Transcatheter Valve Therapy; TAVR=transcatheter aortic valve replacement; VARC-2=Valve Academic Research Consortium 2

In patients with BARC 3C (intracranial, intraocular, etc.), IHB had longer LOS and ICU LOS than in those with BARC 5 (fatal bleeding), and no vascular complications, as patients with BARC 3C bleeding required further treatment and longer monitoring and those with BARC 5 may have died earlier. Most of the patients with VARC-2 major bleeding had access site hematoma or retroperitoneal hematoma, but none of them died.

This study has some limitations. Firstly, it included cases from early phases of the commercial TAVR program in our center. Secondly, as a major referral center, some of our high-or-prohibitive surgical risk patients may have had more complex clinical situations than their counterparts in community hospitals. 

Nevertheless, our study has multiple strengths. It is one of the few studies to focus on IHB; hence, it provides valuable insight to the specific risk factors and outcomes associated during the index hospitalization for TAVR, which is the critical period for bleeding complications. Furthermore, this large cohort reflects a real-world population and provides new knowledge that can contribute to improve patient selection and early planning for prevention and treatment during hospitalization for TAVR.

Patients at higher risk of IHB, such as those with hypoalbuminemia, anemia, and/or planned conduit or transaortic access, may benefit from a more careful selection process and discussions about the higher risk for complications. Further studies should be done on potential interventions to address these risk factors, such as improving nutritional status, treating underlying anemia, etc. These results also suggest that both BARC and VARC-2 definitions may be used to classify the severity of IHB after TAVR and predict outcomes.
